# Atrial Transcriptional Profiles of Molecular Targets Mediating Electrophysiological Function in Aging and *Pgc-1*β Deficient Murine Hearts

**DOI:** 10.3389/fphys.2019.00497

**Published:** 2019-04-24

**Authors:** Charlotte E. Edling, Ibrahim T. Fazmin, Karan R. Chadda, Shiraz Ahmad, Haseeb Valli, Christopher L.-H. Huang, Kamalan Jeevaratnam

**Affiliations:** ^1^Faculty of Health and Medical Sciences, University of Surrey, Guildford, United Kingdom; ^2^Physiological Laboratory, University of Cambridge, Cambridge, United Kingdom; ^3^Department of Biochemistry, University of Cambridge, Cambridge, United Kingdom; ^4^School of Medicine, Perdana University-Royal College of Surgeons in Ireland, Selangor, Malaysia

**Keywords:** peroxisome proliferator activated receptor-γ (PPARγ), coactivator-1 transcriptional coactivator (Pgc-1), quantitative PCR, ion channels, mitochondria, arrhythmias

## Abstract

**Background:**

Deficiencies in the transcriptional co-activator, peroxisome proliferative activated receptor, gamma, coactivator-1β are implicated in deficient mitochondrial function. The latter accompanies clinical conditions including aging, physical inactivity, obesity, and diabetes. Recent electrophysiological studies reported that *Pgc-1*β^-/-^ mice recapitulate clinical age-dependent atrial pro-arrhythmic phenotypes. They implicated impaired chronotropic responses to adrenergic challenge, compromised action potential (AP) generation and conduction despite normal AP recovery timecourses and background resting potentials, altered intracellular Ca^2+^ homeostasis, and fibrotic change in the observed arrhythmogenicity.

**Objective:**

We explored the extent to which these age-dependent physiological changes correlated with alterations in gene transcription in murine *Pgc-1*β^-/-^ atria.

**Methods and Results:**

RNA isolated from murine atrial tissue samples from young (12–16 weeks) and aged (>52 weeks of age), wild type (WT) and *Pgc-1β^-/-^* mice were studied by pre-probed quantitative PCR array cards. We examined genes encoding sixty ion channels and other strategic atrial electrophysiological proteins. *Pgc-1β^-/-^* genotype independently reduced gene transcription underlying Na^+^-K^+^-ATPase, sarcoplasmic reticular Ca^2+^-ATPase, background K^+^ channel and cholinergic receptor function. Age independently decreased Na^+^-K^+^-ATPase and fibrotic markers. Both factors interacted to alter *Hcn4* channel activity underlying atrial automaticity. However, neither factor, whether independently or interactively, affected transcription of cardiac Na^+^, voltage-dependent K^+^ channels, surface or intracellular Ca^2+^ channels. Nor were gap junction channels, β-adrenergic receptors or transforming growth factor-β affected.

**Conclusion:**

These findings limit the possible roles of gene transcriptional changes in previously reported age-dependent pro-arrhythmic electrophysiologial changes observed in *Pgc-1*β^-/-^ atria to an altered Ca^2+^-ATPase (*Atp2a2*) expression. This directly parallels previously reported arrhythmic mechanism associated with p21-activated kinase type 1 deficiency. This could add to contributions from the direct physiological outcomes of mitochondrial dysfunction, whether through reactive oxygen species (ROS) production or altered Ca^2+^ homeostasis.

## Introduction

Atrial arrhythmias constitute a major public health problem. Atrial fibrillation (AF), affects 1–3% of the developed world population. This prevalence will likely increase to ∼9 and ∼18 million cases in the United States and Europe, respectively, by 2060 (Miyasaka et al., 2006). Chronic AF increases incidences of both morbidity, often as stroke, and all-cause mortality.

Age is a major risk factor for AF, affecting 0.1, 4, and 20% of individuals aged <55, 60–70 and >80 years, respectively. So are metabolic factors, arising from physical inactivity, obesity, diabetes mellitus, and metabolic syndrome. Themselves age-dependent, these account for ∼60% of current upward trends in AF incidence (Miyasaka et al., 2006). Both age and metabolic deficiency in turn are associated with mitochondrial dysfunction (Vianna et al., 2006). Age is associated with mitochondrial DNA damage and compromised respiratory chain function (Krishnan et al., 2007); obese mice on high fat diets show defective mitochondrial electron transport chain complex 1 (Wang X. et al., 2015).

Acute and chronic mitochondrial dysfunction have cardiac pro-arrhythmic effects (Akar and O’Rourke, 2011). This indicates abnormalities in one or more of the ordered processes of action potential (AP) initiation, recovery, excitation and propagation. These in turn would reflect altered ion channel function or expression or structural, fibrotic or cardiomyopathic change altering AP conduction, findings reported in experimental diabetes (Russo and Frangogiannis, 2016) and metabolic syndrome (Ho et al., 2017).

Peroxisome proliferator activated receptor-γ (PPARγ) and coactivator-1 transcriptional coactivators (*Pgc-1*) are important cellular energetic regulators. They are abundant in oxidative tissues, including cardiac and skeletal muscle, and brown adipose tissue (Sonoda et al., 2007). They regulate mitochondrial biogenesis and mass (Villena, 2015). They also influence mitochondrial function, increasing expression of genes related to fatty acid β-oxidation, the tricarboxylic acid cycle and electron transport (Arany et al., 2005). Their expression is impaired in metabolic conditions including obesity, insulin resistance, type 2 diabetes and in first-degree relatives of diabetic patients (Scheuermann-Freestone et al., 2003). Pgc-1α levels respond to physiological stimuli such as fasting, exercise and cold temperatures, adapting tissues to changes in energy demand. Pgc-1β appears involved in baseline energetic balance (Villena, 2015).

Finally, Pgc-1α, and Pgc-1β-deficient murine experimental models replicate clinically observed atrial pro-arrhythmic effects of energetic deficiencies. Recent reports implicated age-dependent impairments in chronotropic adrenergic responses, AP generation and conduction despite normal AP recoveries and background resting potentials. They also demonstrated altered intracellular Ca^2+^ homeostasis and fibrotic change (Gurung et al., 2011; Ahmad et al., 2018; Valli et al., 2017a,b, 2018a).

The present studies now explore for transcriptional alterations in the genes encoding the underlying electrophysiological mechanisms in atria of the Pgc-1β-deficient mice (Komen and Thorburn, 2014). Genes were selected and grouped according to the physiological processes underlying different physiological aspects of excitable activity (Huang, 2017). This adapts an approach first applied to rat as opposed to genetically modified mouse hearts (Atkinson et al., 2013). This would make it possible to determine the extent to which previously reported pro-arrhythmic changes reflect altered gene transcription, or whether they more likely follow alterations in subsequent stages in gene expression or even altered function in their resulting proteins.

## Materials and Methods

### Animals

Experiments were approved by the University of Cambridge ethics review board under a United Kingdom project license for studies of cardiac arrhythmia. All procedures complied with United Kingdom Home Office regulations [Animals (Scientific Procedures) Act 1986]. The experiments also conformed to the Guide for the Care and Use of Laboratory Animals, United States National Institutes of Health (NIH Publication No. 85-23, revised 1996). Mice were housed in an animal facility at 21°C with 12-h light/dark cycles. Animals were fed sterile chow (RM3 Maintenance Diet, SDS, Witham, Essex, United Kingdom) and had free access to water, bedding and environmental stimuli. Mice were sacrificed by cervical dislocation. No recovery, anesthetic or surgical procedures were required. Wild Type (WT) C57/B6 and *Pgc-1*β^-/-^ (The Jackson Laboratory, ME, United States) mice were bred for the experimental protocols. Mice were bred on a C57/B6 background to avoid possible strain-related confounds.

### Tissue Samples

Hearts were obtained from four experimental groups, respectively, consisting of young WT, young *Pgc-1*β^-/-^, aged WT, and aged *Pgc-1*β^-/-^ mice. Three hearts were studied from each group. The mice studied were littermates of animals whose hearts had undergone electrophysiological study reported elsewhere (Valli et al., 2017a,b, 2018a,b). Young mice were between the ages of 12–16 weeks old and aged mice greater than 52 weeks of age. After extraction, the heart was cut to separate the atria tissue from the ventricular tissue and both parts were subsequently snap frozen.

### Taqman Array Assay

RNA was extracted from fresh frozen tissues, stored in -80°C, with the Qiagen RNeasy mini Plus kit. Cardiac atrial tissue were weighed and put on ice and subsequently homogenized in RLT buffer supplemented with beta-mercaptoethanol with a Stuart handheld homogenizer until completely smooth. Genomic DNA was eliminated by centrifugation through a column supplied with the kit prior to extraction of the RNA according to the manufacturer’s protocol. RNA integrity was assessed by using an Agilent bioanalyzer to obtain RNA integrity numbers (RIN) according to the manufacturer’s protocol. RNA samples with RINs above 8 were used for the study. The RNA was used to prepare cDNA with High Capacity cDNA Reverse Transcription Kit (Applied Biosystems) according to the manufacturer’s instructions. The efficiency of the cDNA protocol was tested by preparing the cDNA from a serial dilution of the RNA and then these cDNA samples were run on a qPCR confirming equal efficiency over a range of RNA concentrations. cDNA was also confirmed negative for genomic DNA contamination. Each custom made card contained 64 pre-validated assays in triplicate with a reaction volume of 1 μl. The cards were run exactly according to instructions specific for the cards. Briefly, the cDNA (100 ng/well) was mixed with 2× Mastermix from Thermo Fisher Scientific, 100 μl was loaded in each well slot on the cards. The cards were then spun down and sealed and run on a Quant 7 cycler. The amplification conditions were: 50°C for 2 min and 95°C for 10 min for the initial DNA melting and inactivation of the RT reaction, followed by 40 cycles of 95°C for 15 s and 60°C for 60 s. Analysis of the Taqman array card data was performed by using the Quant studio software and Microsoft Excel by calculating fold changes with the ddCT method as described by Livak and Schmittgen (2001). The threshold was set at 0.2 fluorescence units and the baseline range was set to automatic assignment. The geometric mean of the Cq values for the genes *HPRT*, *Gapdh*, and *ActinB* were used as references. *P*-values were calculated using two-way analysis of variance (ANOVA) and Student’s independent *t*-tests.

## Results

The studies explored transcription profiles in atria of young and aged, WT and *Pgc-1*β^-^^/-^ murine hearts previously demonstrated to recapitulate cardiac pro-arrhythmic phenotypes associated with energetic deficiency. Quantitative PCR evaluated transcriptional backgrounds for genes strategic to pro-arrhythmic electrophysiological phenotypes that might in turn constitute potential pharmacological targets (Komen and Thorburn, 2014). Genes were selected and grouped according to the component physiological processes underlying excitable activity leading to arrhythmia (Huang, 2017), adapting approaches introduced in rat hearts (Atkinson et al., 2013). Samples obtained by isolating RNA from murine atrial tissue were studied by qPCR using Thermo Fisher Scientific custom Taqman array cards pre-probed with the 60 different genes of potential interest for cardiac function.

**FIGURE 1:**
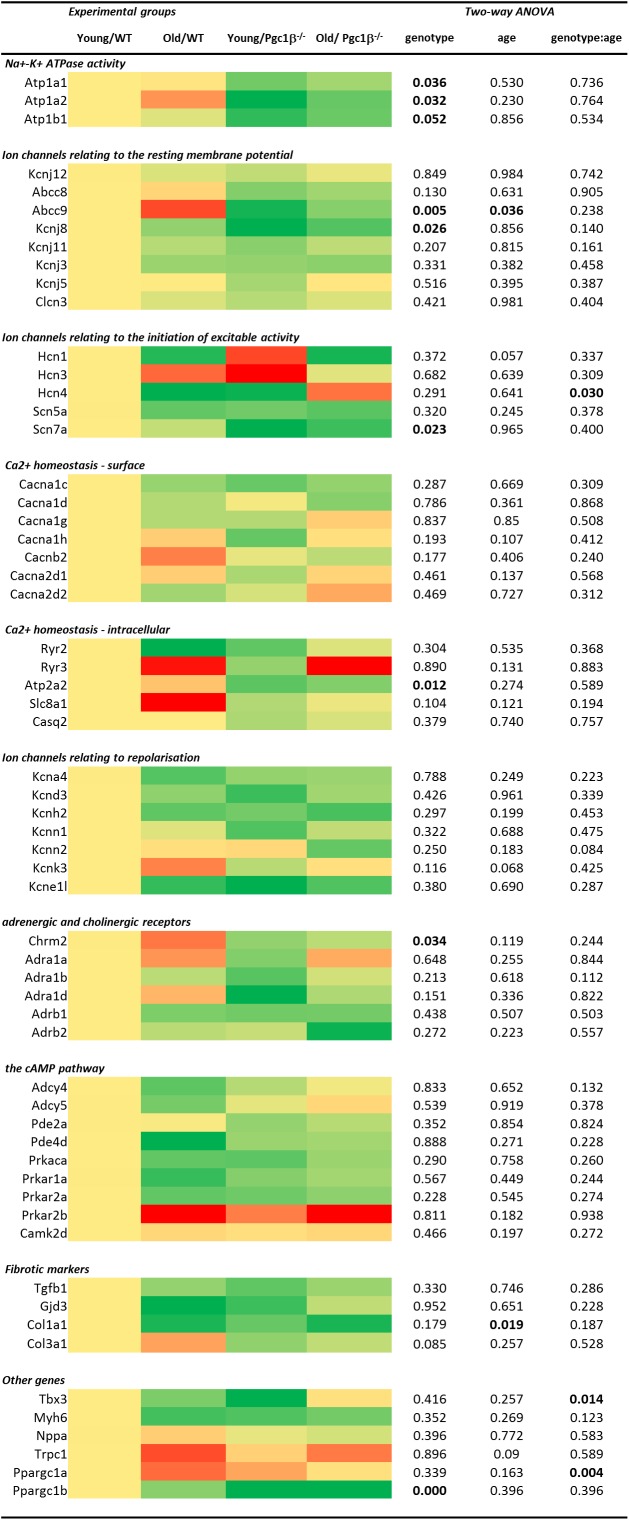
Heatmap visualizing differential expression between the sample groups grouped by electrophysiological function at the individual gene level combined with two-way ANOVA results examining for independent and interacting effects of *Pgc-1*β^-/-^ genotype and age. All fold changes normalized to the mean of the expression in samples from young WT hearts. The heatmap is coded yellow for no change, red for increased and green for decreased in fold gene transcription.

Gene transcription fold changes were normalized to levels for the gene in question in young WT mice (Livak and Schmittgen, 2001). Table 1 lists the genes and the gene expression results obtained, and Figure 1 overviews expression patterns of relative effect sizes and the directions of the observed differences; red backgrounds indicate increased and green decreased expression relative to young WT. Their intensities are scaled to the highest values (darkest red) and lowest values (darkest greens) of the entire set of genes represented in the map with yellow in the middle indicating an absence or little change. These results are accompanied by the results of a two-way analysis of variance testing for independent and interacting effects of genotype and of age to a *P* < 0.05 significance level. Effects of age and genotype were sorted by functional gene group corresponding to the physiological processes described in previous reports on *Pgc-1*β^-^^/-^ phenotypes. Control inclusion of *Ppargc1b* transcription levels confirmed highly significant independent effects of genotype.

**Table 1:** Gene groups examined for transcriptional changes by Taqman PCR in WT and Pgc-1β^-/-^ murine atria.

		WT		WT		Pgc-1β^-/-^		Pgc-1β	
		
		Young		Old		Young		Old	
					
	gene	mean	+/- sem	mean	+/- sem	mean	+/- sem	mean	+/- sem
Na^+^-K^+^ ATPase activity	Atp1a1	1.00	0.07	1.03	0.11	0.72	0.11	0.82	0.09
	Atp1a2	1.00	0.29	1.37	0.24	0.47	0.08	0.70	0.26
	Atp1b1	1.00	0.22	0.94	0.13	0.59	0.04	0.71	0.09
Ion channels relating to the resting membrane potential	Kcnj12	1.00	0.20	0.93	0.20	0.88	0.08	0.96	0.34
	Abcc8	1.00	0.17	1.10	0.05	0.76	0.14	0.82	0.21
	Abcc9	1.00	0.12	1.69	0.21	0.54	0.01	0.76	0.27
	Kcnj8	1.00	0.13	0.79	0.17	0.49	0.08	0.66	0.04
	Kcnj11	1.00	0.13	0.86	0.06	0.77	0.05	0.87	0.05
	Kcnj3	1.00	0.18	0.81	0.11	0.79	0.03	0.78	0.08
	Kcnj5	1.00	0.10	1.00	0.12	0.83	0.01	1.02	0.15
	Clcn3	1.00	0.09	0.93	0.11	0.86	0.04	0.93	0.07
Ion channels relating to the initiation of excitable activity	Hcn1	1.00	0.04	0.57	0.16	1.70	0.69	0.54	0.05
	Hcn3	1.00	0.24	1.56	0.34	2.42	1.77	0.94	0.45
	Hcn4	1.00	0.43	0.32	0.03	0.52	0.23	1.51	0.41
	Scn5a	1.00	0.15	0.69	0.19	0.72	0.11	0.68	0.08
	Scn7a	1.00	0.13	0.88	0.21	0.49	0.10	0.62	0.06
Ca^2+^ homeostasis – surface	Cacna1c	1.00	0.15	0.80	0.13	0.71	0.08	0.79	0.16
	Cacna1d	1.00	0.11	0.85	0.17	0.98	0.29	0.77	0.14
	Cacna1g	1.00	0.30	0.85	0.09	0.85	0.20	1.12	0.48
	Cacna1h	1.00	0.13	1.13	0.14	0.70	0.09	1.05	0.15
	Cacnb2	1.00	0.20	1.46	0.34	0.95	0.14	0.87	0.09
	Cacna2d1	1.00	0.12	1.13	0.12	0.84	0.09	1.10	0.15
	Cacna2d2	1.00	0.41	0.82	0.24	0.92	0.13	1.28	0.12
Ca^2+^ homeostasis – intracellular	Ryr2	1.00	0.13	0.95	0.20	0.69	0.04	0.93	0.18
	Ryr3	1.00	0.25	2.07	1.33	0.79	0.25	2.08	0.25
	Atp2a2	1.00	0.17	1.21	0.11	0.68	0.11	0.75	0.03
	Slc8a1	1.00	0.10	2.16	0.72	0.85	0.07	0.96	0.07
	Casq2	1.00	0.10	1.00	0.21	0.84	0.10	0.92	0.03
Ion channels relating to repolarisation	Kcna4	1.00	0.14	0.67	0.08	0.79	0.08	0.80	0.19
	Kcnd3	1.00	0.31	0.78	0.13	0.62	0.06	0.82	0.23
	Kcnh2	1.00	0.23	0.69	0.14	0.73	0.07	0.64	0.07
	Kcnn1	1.00	0.22	0.94	0.27	0.66	0.07	0.88	0.13
	Kcnn2	1.00	0.08	1.06	0.14	1.08	0.09	0.70	0.12
	Kcnk3	1.00	0.18	1.45	0.13	0.86	0.16	1.05	0.13
	Kcne1l	1.00	0.28	0.60	0.16	0.47	0.02	0.66	0.39
Adrenergic and cholinergic receptors	Chrm2	1.00	0.17	1.49	0.26	0.79	0.05	0.87	0.08
	Adra1a	1.00	0.21	1.37	0.56	0.75	0.15	1.27	0.38
	Adra1b	1.00	0.05	0.87	0.15	0.67	0.06	0.91	0.12
	Adra1d	1.00	0.27	1.23	0.48	0.48	0.09	0.84	0.12
	Adrb1	1.00	0.24	0.75	0.21	0.72	0.10	0.73	0.15
	Adrb2	1.00	0.27	0.86	0.19	0.89	0.18	0.52	0.06
The cAMP pathway	Adcy4	1.00	0.07	0.79	0.10	0.86	0.09	0.98	0.13
	Adcy5	1.00	0.23	0.82	0.11	0.95	0.20	1.09	0.12
	Pde2a	1.00	0.18	0.99	0.14	0.79	0.16	0.86	0.20
	Pde4d	1.00	0.09	0.67	0.04	0.80	0.11	0.82	0.22
	Prkaca	1.00	0.18	0.79	0.06	0.68	0.12	0.80	0.15
	Prkar1a	1.00	0.17	0.74	0.05	0.76	0.16	0.82	0.09
	Prkar2a	1.00	0.14	0.79	0.15	0.72	0.08	0.78	0.07
	Prkar2b	1.00	0.23	4.84	2.44	1.46	0.65	5.75	4.94
	Camk2d	1.00	0.08	1.35	0.25	1.06	0.07	1.09	0.04
Fibrotic markers	Tgfb1	1.00	0.15	0.79	0.15	0.69	0.06	0.80	0.18
	Gjd3	1.00	0.45	0.46	0.04	0.62	0.33	0.88	0.26
	Col1a1	1.00	0.09	0.55	0.09	0.70	0.15	0.55	0.05
	Col3a1	1.00	0.16	1.31	0.07	0.78	0.24	0.88	0.15
Other genes	Tbx3	1.00	0.17	0.74	0.05	0.46	0.12	1.05	0.16
	Myh6	1.00	0.17	0.63	0.11	0.66	0.09	0.73	0.12
	Nppa	1.00	0.13	1.12	0.21	0.95	0.08	0.92	0.10
	Trpc1	1.00	0.24	1.67	0.15	1.12	0.32	1.49	0.33
	Ppargc1a	1.00	0.03	1.55	0.13	1.30	0.10	1.05	0.10
	Ppargc1b	1.00	0.15	0.77	0.21	0.00	0.00	0.00	0.00


Of genes related to (1) the energetically dependent Na^+^-K^+^-ATPase-mediated membrane transport required to generate ionic gradients upon which excitable activity depends and (2) background K^+^ channels mediating the resting potential, we tested transcription activity for Na^+^-K^+^ ATPase α_1_ and α_2_ catalytic and accessory β_1_ subunits (encoded by *Atpa1*, *Atpa2*, and *Atpb1*, respectively), the ATP-sensitive inward rectifier K^+^ channel Kir2.2 (*Kcnj12*) coupling resting potentials to intracellular nucleotide levels (Nichols, 2006), and the ATP-binding cassette (ABC) transporter subunits members 8 and 9 (*Abcc8* and *Abcc9*), inwardly rectifying pore-forming K^+^ channels Kir6.1, Kir6.2, and Kir3.1 (*Kcnj8*, *Kcnj11*, and *Kcnj3*) and G protein-activated inward rectifier potassium channel 4, Kir3.4 (*Kcnj5*). The two-way ANOVA suggested independent effects of genotype decreasing *Atp1a1*, *Atp1a2*, *Atp1b1, Kcnj8*, and *Abcc9* transcription, and independent effects of age decreasing *Abcc9* transcription.

Of (3) voltage-dependent activating processes, the two-way ANOVA suggested independent effects of genotype decreasing *Scn7a* and interacting effects of genotype and age altering expression of *Hcn4* known to mediate hyperpolarization-activated cyclic nucleotide–gated channels underlying SAN pacemaker currents I_f_ (Thollon et al., 2007). It did not detect altered transcription of the major, *Scn5a*, isoform concerned with cardiac Na^+^ current. Nor did the two-way ANOVA demonstrate either independent or interacting effects of genotype and age on (4) the surface membrane voltage-dependent L-type Ca^2+^ channels Cav1.2 and Cav1.3 (*Cacna1c* and *Cacna1d*), T-type, Cav3.1 (*Cacna1g*), and Cav3.2 (*Cacna1h*), and the accessory β2 (*Cacnb2*), α_2_/δ1 (*Cacna2d1*) and α_2_/δ_2_ subunits (*Cacna2d2*). Of (5) molecules related to intracellular Ca^2+^ homeostasis between cellular compartments, there were neither independent nor interacting effects on transcription of ryanodine receptor isoforms RyR2 (*Ryr2*) and RyR3 (*Ryr3*), the principal cardiac Na^+^-Ca^2+^ exchanger (*Slc8a1*), and cardiac SR Ca^2+^ binding protein (*Casq2*). However, there was a significant effect of genotype upon the transport mediating Ca^2+^-ATPase (*Atp2a2*).

The two-way ANOVA revealed no significant effects bearing on (6) transcription on voltage dependent K^+^ channels underlying AP recovery, including voltage sensitive transient outward current I_to_, Kv1.4 (*Kcna4*), and Kv4.3 (*Kcnd3*), rapid K^+^ current, I_Kr_, Kv11.1 (*Kcnh2*), the recently characterized Ca^2+^-activated K^+^ channel K_Ca_2.1 (*Kcnn1*) and K_Ca_2.2 (*Kcnn2*) that have a selective atrial as opposed to ventricular occurrence thought to contribute to AP (Xu et al., 2003). This was also the case for the two-predomain TWIK-related acid-sensitive potassium channel 1, TASK-1/K 2P 3.1 (*Kcnk3*) (Olschewski et al., 2017) and the regulatory KCNE1 subunit (*Kcne11*).

Of (7) receptor proteins related to cardiomyocyte cholinergic and adrenergic autonomic activation, the two-way ANOVA detected an independent effect of genotype decreasing muscarinic M_2_ (*Chrm2*) expression. There were no effects upon the three cardiac, α_1A_, α_1B_, and α_1D_ (*Adra1a*, *Adra1b*, and *Adra1d*) adrenergic receptor subtypes, thought to protect against pathological remodeling in heart failure (O’Connell et al., 2013) or β_1_ and β_2_- adrenergic receptor subtypes (*Adrb1* and *Adrb2*). The two-way ANOVA detected no effects on transcription of (8) cyclic AMP-dependent cellular messenger pathways involved in such autonomic signaling of which we explored cardiac adenylyl cyclase, types 4 and 5 (*Adcy4* and *Adcy5*), of which type 5 accentuates cardiomyopathic changes on chronic catecholamine stimulation, cGMP-dependent and cAMP-specific 3′,5′-cyclic phosphodiesterases 2A and 4D (*Pde2a* and *Pde4d*), and the protein kinase A catalytic α-subunit (*Prkaca*). We did not observe altered transcription in cardiac Ca^2+^/calmodulin-dependent protein kinase, type II-δ (*Camk2d*) (Zhang et al., 2004).

The only alterations suggested for markers for (9) atrial fibrosis, concerned independent effects of age in reducing *Col1a1*, the major component of type I collagen, the fibrillar collagen found in most connective tissues. Transcription of the remaining cytokine transforming growth factor β (TGF-β1; *Tgfb1*) (Hao et al., 2011), connexin mCx30.2 (*Gjd3*) (Kreuzberg et al., 2005), and the collagen precursor for the collagen type III α1 chain (*Col3a1*) (Davies et al., 2014) were largely unchanged. The final set of genes tested are thought to act as (10) markers for a range of developmental, inflammatory and hypertrophic changes ultimately impacting AP generation and propagation, as well as the genes encoding *Pgc-1*β itself and the complementary *Pgc-1α.* Translation of both the transcriptional repressor known to affect particular components of the cardiac conduction system, *Tbx3* (Sylva et al., 2014), and the major thick filament protein *Myh6* (MHC-α) whose mutations are associated with late-onset hypertrophic cardiomyopathy, atrial septal defects and sinus node disorder (Huang, 2017). Mutations in atrial natriuretic peptide (Nppa) have been implicated in familial AF (Perrin and Gollob, 2012). The non-specific ion channel *Trpc1* conducts both Ca^2+^ and Na^+^ with Ca^2+^ store depletion or activation of the phospholipase C system with associations with hypertrophic change showed increases in groups relative to young WT (Xu and Beech, 2001). The two-way ANOVA did not demonstrate any effects of age or genotype apart from interacting effects on the gene *Pgc-1*α gene known to complement the function of *Pgc-1*β^-^^/-^ and on the gene *Tbx3*.

**FIGURE 2:**
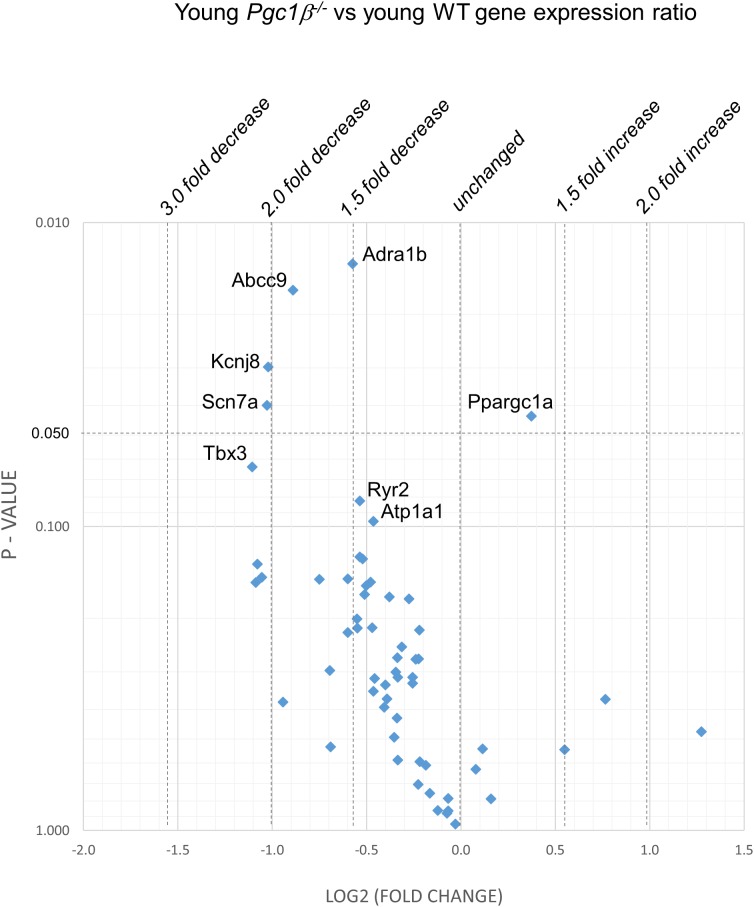
Volcano plot of differentially expressed genes comparing young *Pgc-1*β^-/-^ with young WT. Plots of stratified *P*-values against expression fold changes for results expressing young *Pgc-1*β^-/-^ normalized to young-WT.

**FIGURE 3:**
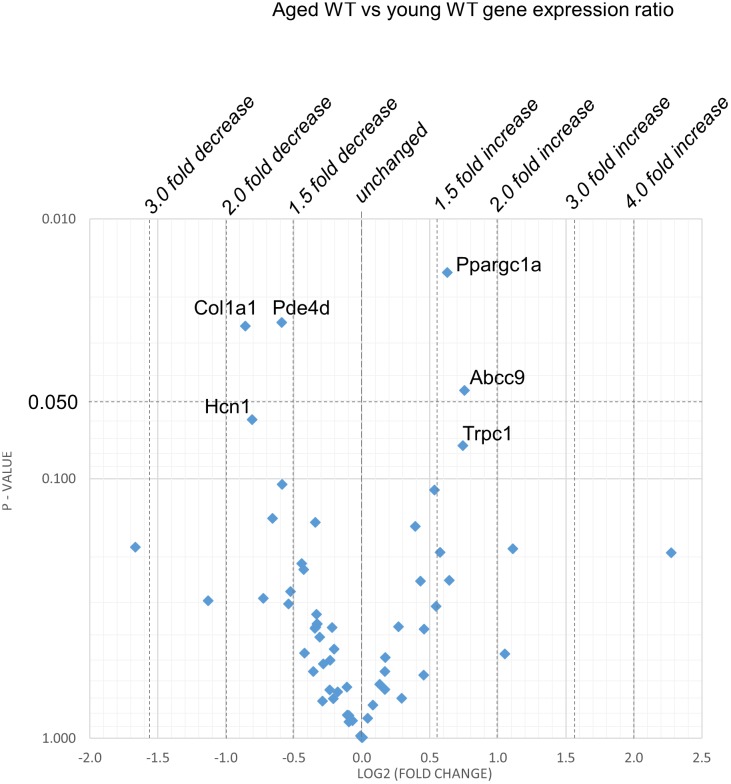
Effect of aging in the WT: volcano plot of differentially expressed genes comparing aged WT with young WT. Plots of stratified *P*-values against expression fold changes for results expressing aged WT normalized to young WT.

**FIGURE 4:**
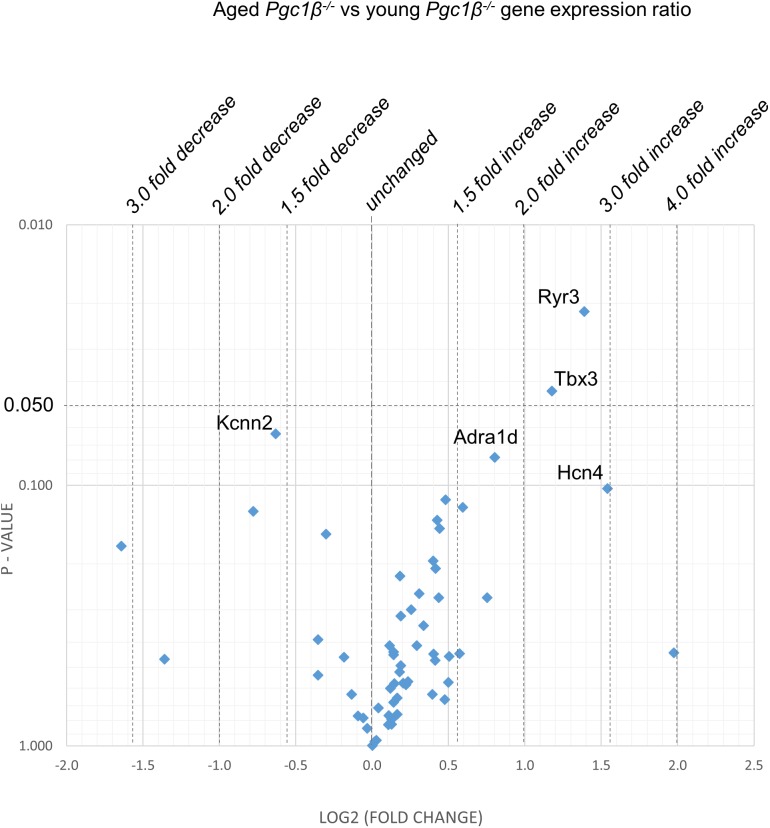
Effect of aging in the *Pgc-1*β^-/-^: volcano plot of differentially expressed genes comparing aged *Pgc-1*β^-/-^ with young *Pgc-1*β^-/-^. Plots of stratified *P*-values against expression fold changes for results expressing aged *Pgc-1*β^-/-^ normalized to young *Pgc-1*β^-/-^.

**FIGURE 5:**
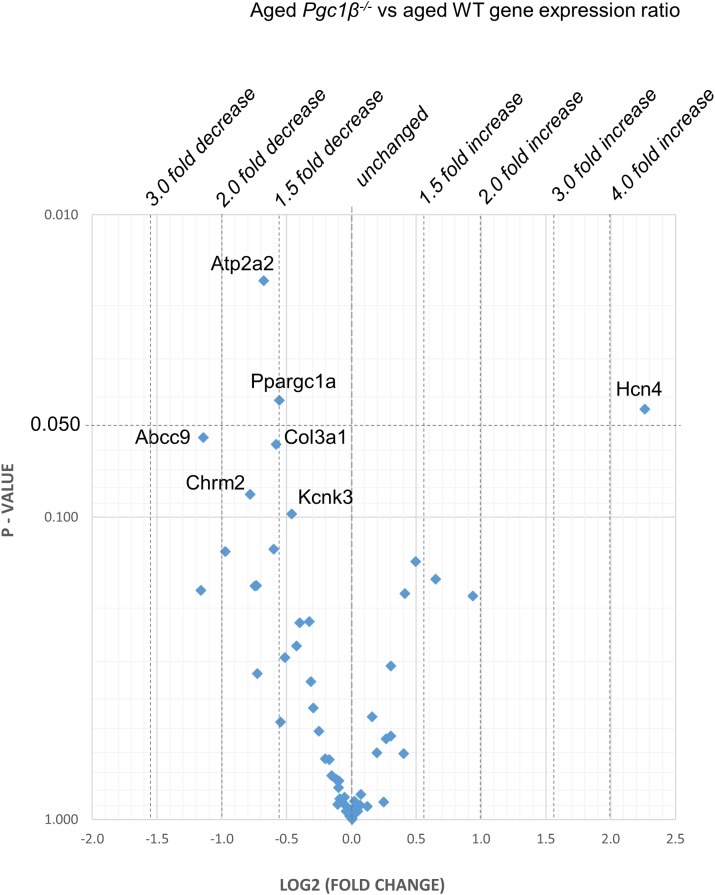
Aged *Pgc-1*β^-/-^ compared with aged WT: volcano plot of differentially expressed genes comparing young *Pgc-1*β^-/-^ with young WT. Plots of stratified *P*-values against expression fold changes for results expressing *Pgc-1*β^-/-^ normalized to young WT.

Figure 2–5 display volcano plots derived from statistical assessments of transcriptional differences in individual genes between atria from young and aged, WT and *Pgc-1*β^-/-^ mice stratified by *P*-values (<0.01, <0.05 and <0.10, respectively) and plotted against their effect sizes. Comparisons were made between young *Pgc-1*β^-/-^ and young WT (Figure 2), the effects of aging in WT (Figure 3) and *Pgc-1*β^-/-^ (Figure 4), respectively, and the eventual differences between aged *Pgc-1*β^-/-^ and aged WT (Figure 5). They thus provide graphical indications as to where the significant paired differences were to be found, plotting the data points in their entirety over the full range of their *P*-values, and identifying genes where they had *P* < 0.1. In all these comparisons, there were no alterations in transcription of genes bearing on Nav1.5, L or T-type Ca^2+^ channel, Ca^2+^ homeostasis, or repolarizing K^+^ channel function. This was with the exception of Atp2a2 in Figure 5, and Ryr3 in Figure 4. Of the remaining genes analyzed:

(1) Compared to young WT hearts, young *Pgc-1*β^-/-^ demonstrated (*P* < 0.05) reduced transcription in the following genes concerned with resting potential maintenance (Figure 2): inwardly rectifying Kir6.1 (*Kcnj8*) and regulatory subunits of the ATP-sensitive K^+^ channel 9 (*Abcc9*). There was also a small though significant *Ppargc1a* increase suggesting a compensation for the reduced *Ppargc1b* transcription. Of genes bearing on activation of electrophysiological activity, *Scn7a*, but not the principal *Scn5a* isoform, showed altered transcription. Of molecules underlying intracellular Ca^2+^ homeostasis, the trend (*P* < 0.1) toward reduced cardiac RyR2-Ca^2+^ SR channel (*Ryr2*) transcription was in a direction contrasting with previously reported increased SR Ca^2+^ release phenotypes (Gurung et al., 2011). Patterns of β-adrenergic receptor subtype transcription showed no change, in contrast with the previously reported chronotropic incompetence associated with *Pgc*-1β^-/-^ (Lelliott et al., 2006). Similarly, there were no alterations in markers for fibrotic change (*Tgfb1*, *Gjd3*, *Col1a1*, and *Col3a1*), although there was a reduced transcription of α_1B_-adrenergic receptor subtypes (*Adra1b*) known to mediate protective and adaptive functions preventing pathological remodeling in cardiac failure through Gq/11 signaling (O’Connell et al., 2013) and of the transcriptional repressor *Tbx3*. There was increased transcription of the complementary Pgc-1α (*Ppargc1a*) involved in adjustments to altered metabolic demand.
(2) Figure 3 explores the effects of aging in WT atria. There was again a *Ppargc1a* increase compensating for the reduced *Ppargc1b* transcription. In contrast to the comparisons between young *Pgc-1*β^-/-^ and young WT, aging increased (*P* < 0.05) transcription of the ATP-sensitive K^+^ channel 9 regulatory subunit (*Abcc9*), concerned with resting potential maintenance. The trend toward reduced transcription in HCN channels only concerned *Hcn1* rather than the principal *Hcn4* isoform. A decreased *Pde4d* proved to be exclusive to the WT mice, and consequently had not been detected when analyzed with the *Pgc-1*β^-/-^ in the ANOVA.
  However, there were no changes concerning Nav1.5, molecules related to L or T-type Ca^2+^ channel function or repolarising K^+^ channels, or of RyR2 or other molecules controlling intracellular Ca^2+^ homeostasis. There was increased transcription of the complementary Pgc-1α (*Ppargc1a*) involved in adjustments to altered metabolic demand. Transcription of the *Col1a1* marker for fibrotic change was reduced. There was a trend toward decreased transcription of TRPC channels involved in signaling cascades mediating cardiac hypertrophy and remodeling (Eder and Molkentin, 2011; Freichel et al., 2017) was increased.
(3) Figure 4 illustrates effects of aging in *Pgc-1*β^-/-^ suggestive of particular effects of *Pgc-1*β^-/-^ on age-related remodeling changes. Aging *Pgc*-1β^-/-^ showed features not shown by aging WT: there was increased transcription of the RyR3 ryanodine receptor isoform (*Ryr3*) and *Tbx3*, and trends toward increased transcription of the principal HCN4 variant of the channels concerned with sinoatrial pacemaker current. There was also trends toward decreased transcription of KCa2.2 (*Kcnn2*) and increased transcription of adrenergic receptor subtype *Adra1d*, thought to protect against pathological remodeling in heart failure. Aging *Pgc-1*β^-/-^ did not show the decreased translation of *Col1a1* or *Abcc9* in contrast to aging WT.
(4) Figure 5 compares transcriptional profiles in aged *Pgc*-1β^-/-^ with those of aged WT constituting the end situations arising from the processes represented in Figure 3, 4. This revealed a *Ppargc1a* decrease in contrast to its increase in some of the remaining comparisons. In aged *Pgc-1*β^-/-^ compared to aged WT, there was a markedly increased transcription of the principal *Hcn4* isoform concerned with sinoatrial pacemaker current and decreased transcription of the SR Ca^2+^-ATPase subunit *Atp2a2* and of *Pgc*-1α (*Ppargc1a*). There were trends toward decreased transcription of the type I collagen marker *Col3a1*, TWIK-related acid-sensitive potassium channel 1 (*Kcnk3*), the ATP-sensitive K^+^ channel 9 (*Abcc9*), and the cholinergic M2 receptor (*Chrm2*).

## Discussion

The present studies applied quantitative PCR to explore for age-dependent transcriptional changes in genes strategic to electrophysiological phenotypes underlying atrial pro-arrhythmic tendency in murine hearts deficient in the transcriptional coactivator peroxisome proliferator activated receptor-γ coactivator-1β (PGC-1β). *Pgc-1*β^-/-^ hearts have proved useful experimental models in studies of atrial arrhythmogenic mechanisms associated with clinical conditions arising from energetic, particularly mitochondrial, functional deficiencies. Of these, the most widespread are atrial arrhythmias, particularly AF for which age and metabolic factors constitute major risk factors through their associations with mitochondrial dysfunction and its associated impaired oxidative capacity (Vianna et al., 2006). Pgc-1α and Pgc-1β offer potential therapeutic targets. The PPAR-α agonist fenofibrate, inhibited atrial metabolic remodeling in AF (Liu W.Y. et al., 2016; Zhao et al., 2016). Rosiglitazone reduced atrial interstitial fibrosis and AF promotion in diabetic rabbits by modulating oxidative stress and inflammation (Lin et al., 2014). The specific PPARγ ligands the thiazolidinediones (TZDs) improve clinical insulin sensitivity in type 2 diabetes mellitus (Liu A. et al., 2016). They may also decrease myocardial fibrosis and improve cardiac function (Liu A. et al., 2016).

Recent electrophysiological studies had demonstrated age-dependent atrial arrhythmic phenotypes in murine *Pgc-1*β^-/-^ hearts relating these to abnormal electrophysiological properties, Ca^2+^ homeostasis and fibrotic change (Valli et al., 2017a,b, 2018a; Ahmad et al., 2018). The present quantitative PCR studies went on to explore for altered transcription of genes selected to encode the ion channels, other transport proteins, markers for autonomic and energetic function and developmental, inflammatory, and fibrotic and hypertrophic changes that may underly those reported physiological changes. It was therefore possible to determine whether the electrophysiological differences (Valli et al., 2017a,b, 2018a; Ahmad et al., 2018) correlated with correspondingly altered patterns of protein transcriptional change, or otherwise. Whilst not distinguishing surface from internal membrane protein expression, they revealed differing patterns of transcriptional change in proteins related to each process. This approach had previously successfully been applied to studies in rat as opposed to genetically modified mouse hearts (Atkinson et al., 2013). Thus the previous electrophysiological studies in young and old, WT and *Pgc-1*β^-/-^, hearts reported:

(1) Impaired sino-atrial pacing and negative dromotropic responses suggesting defective atrioventricular conduction following adrenergic activation particularly in intact aged *Pgc-1*β^-/-^ mice (Ahmad et al., 2018) and impaired chronotropic responses to adrenergic stimulation (Lelliott et al., 2006). In contrast, 2-way ANOVA detected no significant differences between groups in transcription of autonomic β-adrenergic receptors, but did detect genotypic effects decreasing the cholinergic *Chrm2*. There were no differences in HCN4 transcription mediating pacemaker currents. Differences suggested by the volcano plots did not bear on β-adrenergic responses; young *Pgc-1*β^-/-^ showing lower *Adra1b* expression than young WT, and old WT showing lower *Pde4d* expression than young WT. Aged *Pgc-1*β^-/-^ showed increased *Hcn4* expression compared to aged WT, contrasting with the compromised SAN response to adrenergic challenge shown by *Pgc-1*β^-/-^.

In intact Langendorff-perfused hearts, there were (2) similar resting potentials between experimental groups (Valli et al., 2017b). Yet markers for molecules concerned with resting potential generation indicated reduced transcription in several genes related to resting potential. The two-way ANOVA demonstrated that the *Pgc-1*β*^-/-^* genotype decreased the markers of Na^+^-K^+^-ATPase transcription *Atp1a1*, *ATP1a2*, and *Atp1b1*, and those of the K+ channels Abcc9 and Kcnj8. The volcano plots indicated that young *Pgc-1*β*^-/-^* showed reduced *Abcc9* and *Kcnj8* relative to young WT, and aged WT, increased *Abcc9* relative to young WT.

(3)-(6) *Pgc-1*β*^-/-^* showed (3) age-dependent slowing of AP conduction associated with reduced AP upstroke rates (d*V*/d*t*)_max_ implicating reduced Na^+^ channel function (Valli et al., 2017a). In contrast, (4) AP repolarization rates reflected in action potential durations (APDs) remained similar between experimental groups (Valli et al., 2017b). Loose-patch clamp studies related the above findings to (5) reduced depolarizing, Na^+^, but (6) normal repolarizing voltage-dependent and inward rectifier K^+^ current in both young and aged *Pgc-1*β^-/-^ genotype (Valli et al., 2018a). In contrast to (3) and (5), both two-way ANOVA and volcano plots demonstrated no differences between groups for Nav1.5 (*Scn5a*), or surface L- or T-type Ca^2+^ channel related genes relevant to initiation or maintenance of AP depolarization. However, in accord with (4) and (6), markers for voltage-dependent K^+^ channels underlying AP repolarization duration were indistinguishable between groups culminating in indistinguishable outcomes between aged *Pgc-1*β^-/-^ and aged WT.

Finally, *Pgc-1*β^-/-^ was associated with (7) *Ca^2+^ homeostatic changes* reflected in diastolic SR Ca^2+^ release events, altered Ca^2+^ current characteristics and consequent early and delayed afterdepolarization phenomena (Gurung et al., 2011). In contrast, both two-way ANOVA and the volcano plots revealed no systematic changes in markers for Ca^2+^ homeostatic change mediating RyR2 (*Ryr2*), Na^+^/Ca^2+^ exchange (*Slc8a1*) or calsequestrin (*Casq2*). However, there were independent genotypic effects on sarcoplasmic reticular Ca^2+^ ATPase (*Atp2a2*). These manifested as diminished *Atp2a2* transcription in old *Pgc-1*β^-/-^ compared to aged WT (but not young *Pgc-1*β^-/-^) hearts, with increased expression of Ryr3 (but not Ryr2) in old *Pgc-1*β^-/-^ compared to young *Pgc-1*β^-/-^.

(8) The *Pgc-1*β*^-/-^* genotype also was responsible for producing an age-dependent fibrotic change itself contributing to slowed AP conduction (Jeevaratnam et al., 2011). In contrast, both two-way ANOVA and volcano plots demonstrated that age independently reduced expression of *Col1a1* though there were no effects whether independent or interacting of *Pgc-1*β^-/-^ in the remaining (*Tgfb1*, *Gjd5*, *Col3a1)* transcription markers. However, young *Pgc-1*β*^-/-^* showed reduced α_1B_-adrenergic receptor (*Adra1b*) transcription compared to young WT known to mediate protective and adaptive functions preventing pathological remodeling in cardiac failure through Gq/11 signaling (O’Connell et al., 2013). Of examined markers for a range of developmental, inflammatory and hypertrophic changes, two-way ANOVA demonstrated no differences bearing on *Tbx3, Myh6*, or *Nppa* although *Tbx3* was increased in aged relative to young *Pgc-1*β^-/-^.

Finally, two way ANOVA demonstrated that both genotype and age independently and interactingly exerted transcription changes in the complementary *Pgc-1*α gene (*Ppargc1a*). There was increased transcription in aged WT compared to young WT, and in young *Pgc-1*β*^-/-^*compared to young WT but decreased transcription in aged *Pgc-1*β*^-/-^* compared to aged WT.

The present findings together limit roles for transcriptomic changes in the age-dependent pro-arrhythmic phenotypic features of *Pgc-1*β^-/-^ to altered Ca^2+^-ATPase (*Atp2a2*) expression. However, this is compatible with a previously reported arrhythmic mechanism demonstrated in p21-activated kinase type 1 deficient hearts on an earlier occasion (Wang et al., 2014; Wang Y. et al., 2015). These could add to contributions from direct physiological consequences of altered mitochondrial dysfunction. Thus the latter increases reactive oxygen species (ROS) production that acutely affects voltage-dependent Na^+^ and K^+^ channels (Wang et al., 2004; Liu et al., 2010), ryanodine receptors and gap junctions (Terentyev et al., 2008; Brown and O’Rourke, 2010; Sovari et al., 2013). Both transcriptomically and physiologically produced alterations in Ca^2+^ homeostasis could reduce Nav1.5 function (King et al., 2013; Ning et al., 2016), in turn associated with increased TGF-β activity, fibrotic change (Brooks and Conrad, 2000; Rosenkranz et al., 2002; Dai et al., 2009; Hafner et al., 2010; Hao et al., 2011), and disrupted gap junction function (van Veen et al., 2005; Chilton et al., 2007; Xie et al., 2009). Both Nav1.5 and gap junction changes may contribute pro-arrhythmic conduction changes observed in *Pgc-1*β^-/-^ hearts.

## Ethics Statement

Experiments were approved by the University of Cambridge ethics review board under a United Kingdom project license for studies of cardiac arrhythmia. All procedures complied with the United Kingdom Home Office regulations [Animals (Scientific Procedures) Act 1986]. The experiments also conformed to the Guide for the Care and Use of Laboratory Animals, United States National Institutes of Health (NIH Publication No. 85-23, revised 1996).

## Author Contributions

CE, KC, and IF performed the genome transcription studies, HV and SA performed the specimen preparation, CH and KJ conceived and designed the study and wrote the manuscript.

## Conflict of Interest Statement

The authors declare that the research was conducted in the absence of any commercial or financial relationships that could be construed as a potential conflict of interest.
